# Microaerobic metabolism of lactate and propionate enhances vitamin B_12_ production in *Propionibacterium freudenreichii*

**DOI:** 10.1186/s12934-022-01945-8

**Published:** 2022-10-28

**Authors:** Alexander Dank, Gabriela Biel, Tjakko Abee, Eddy J. Smid

**Affiliations:** Food Microbiology, Wageningen University and Research, Wageningen, The Netherlands

**Keywords:** *Propionibacterium freudenreichii*, Vitamin B_12_, Respiration, Propionate, Wood-Werkman cycle

## Abstract

**Background:**

*Propionibacterium freudenreichii* is used in biotechnological applications to produce vitamin B_12_. Although cultured mainly in anaerobic conditions, microaerobic conditions can greatly enhance biomass formation in *P. freudenreichii*. Since B_12_ yields may be coupled to biomass formation, microaerobic conditions show great potential for increasing B_12_ yields in *P. freudenreichii*.

**Results:**

Here we show biomass formation increases 2.7 times for *P. freudenreichii* grown in microaerobic conditions on lactate versus anaerobic conditions (1.87 g/L vs 0.70 g/L). Consumption of lactate in microaerobic conditions resulted first in production of pyruvate, propionate and acetate. When lactate was depleted, pyruvate and propionate were oxidised with a concomitant sixfold increase in the B_12_ titer compared to anaerobic conditions, showing potential for propionate and pyruvate as carbon sources for B_12_ production. Consequently, a fed-batch reactor with anaerobically precultured lactate-grown cells was fed propionate in microaerobic conditions resulting in biomass increase and production of B_12_. Vitamin yields increased from 0.3 $\mu g$ B_12_ per mmol lactate in anaerobic conditions to 2.4 $\mu g$ B_12_ per mmol lactate and 8.4 $\mu g$ B_12_ per mmol propionate in microaerobic conditions. Yield per cell dry weight (CDW) increased from 41 $\mu g$ per g CDW in anaerobic conditions on lactate to 92 $\mu g$ per g CDW on lactate and 184 $\mu g$ per g CDW on propionate in microaerobic conditions.

**Conclusions:**

Here we have shown both B_12_ yield per substrate and per CDW were highest on cells oxidising propionate in microaerobic conditions, showing the potential of propionate for biotechnological production of vitamin B_12_ by *P. freudenreichii.*

**Supplementary Information:**

The online version contains supplementary material available at 10.1186/s12934-022-01945-8.

## Background

Vitamin B_12_ (B_12_), or cobalamin, is an essential vitamin for humans which is exclusively produced by some Bacteria and Archaea. It acts as a co-factor in enzymatic processes, which can be divided into carbon rearrangement reactions, intramolecular methyl transfer reactions and reduction of ribonucleotide triphosphate to 2-deoxyribonucleotide triphosphate [1]. In propionic acid bacteria B_12_ acts as a co-factor in the characteristic Wood-Werkman cycle used to ferment substrates such as lactate. B_12_ is essential in the isomerisation of succinyl-CoA to methylmalonyl-CoA [2], as it acts as a co-factor of methylmalonyl-CoA mutase. B_12_ thus plays an essential role in the main metabolism of propionic acid bacteria under anaerobic fermentation conditions.

B_12_ can be synthesised in bacteria through an aerobic and an anaerobic pathway, of which the anaerobic pathway is used by *Propionibacterium freudenreichii* [3, 4]. Although the B_12_ production pathway in *P. freudenreichii* is anaerobic, yield increments have been reported for *P. freudenreichii* grown under aerobic conditions [5]. On the other hand, Quesada-Chanto et al. [6] and Menon et al. [7] found decreased B_12_ production when oxygen was present. The presented studies on B_12_ production have in common that relatively high amounts of oxygen are used, resulting in decreased cytochrome synthesis [5, 8] potentially caused by diminished $\delta$-aminolevulinic acid dehydratase activity [7], resulting in lower growth rates and at higher oxygen levels even in diminished growth. Since heme and B_12_ share the same precursors produced by $\delta$-aminolevulinic acid dehydratase, a decreased B_12_ yield could be expected when oxygen diminishes the respective dehydratase activity.

Recently Dank et al. [9] have shown lactate can be completely oxidised using a continuous flow of low amounts of oxygen in a three phase cultivation. Under these conditions, large proportions of lactate are fermented to propionate and acetate, after which, when lactate is depleted, propionate starts being oxidised and lastly acetate is being oxidised. The production and subsequent consumption of propionate shown by Dank et al. [9] can be explained by operation of the Wood-Werkman cycle in reverse direction [10] and a functional electron transport chain. The terminal oxidase of *P. freudenreichii* in the electron transport chain is cytochrome bd [11], which contains heme [12]. Since a functional electron transport chain is required for oxidising propionate with oxygen as terminal electron acceptor, it is conceivable that the conditions used by Dank et al. [9] allow heme, and thus also B_12_ synthesis. As B_12_ is required as co-factor for methylmalonyl-CoA mutase, reversing the Wood-Werkman cycle conceivably still results in a metabolic demand for B_12_ and consequently B_12_ production. This led us to test the hypothesis that B_12_ is actively produced by *P. freudenreichii* utilising propionate. In this study we confirm the ‘propionate switch’ observed by Dank et al. [9] in microaerobic conditions on lactate and consequently show microaerobic conditions enhance B_12_ yield on lactate. Furthermore we show propionate can be used as sole carbon source for the production of B_12_ and we show B_12_ yields are drastically improved using propionate as sole carbon source under microaerobic conditions compared to lactate in microaerobic and anaerobic conditions.

## Results

### Biomass formation and B_12_ yield on lactate drastically increase in microaerobic conditions

Biomass formation and B_12_ yield were monitored in *P. freudenreichii* cultures grown on lactate in anaerobic and microaerobic conditions.

**Fig. 1:**
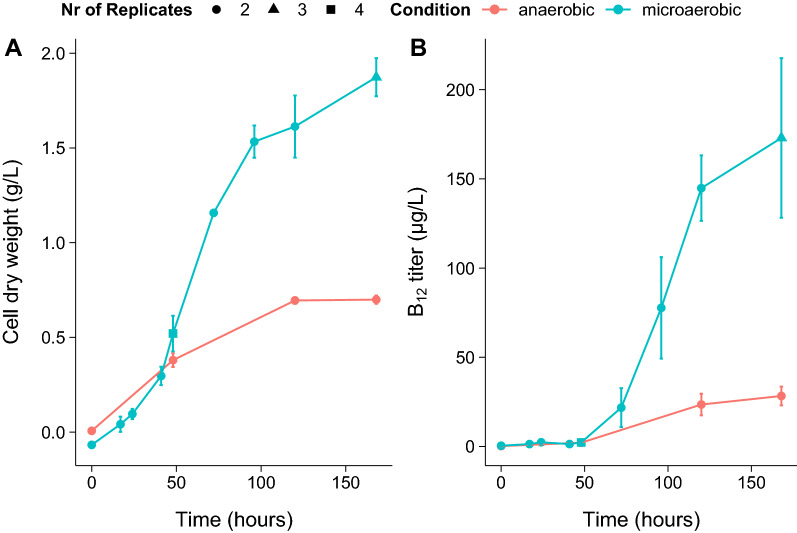
Biomass formation (**A**) and B_12_ titer (**B**) in anaerobic and microaerobic conditions for growth of *P. freudenreichii* on lactate. Error bars represent standard error from biological replicates. Number of replicates per timepoint are displayed by circles (n = 2), triangles (n = 3) or squares (n = 4)

**Fig. 2:**
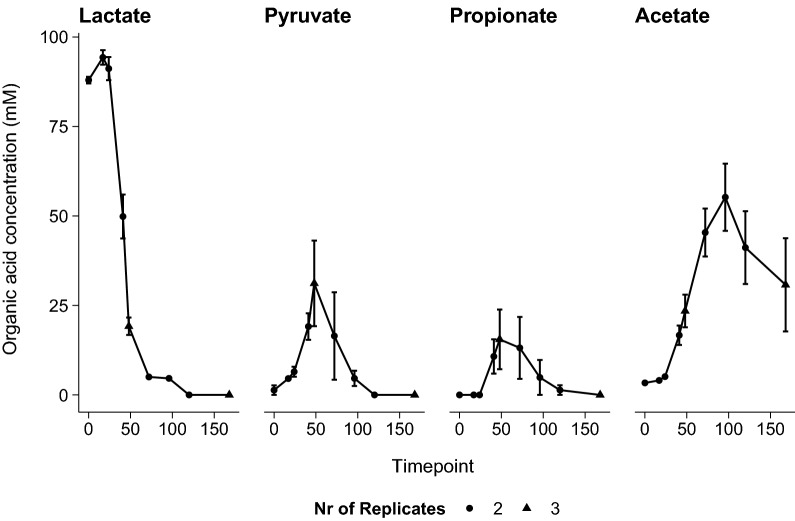
Substrate consumption and primary metabolite production in microaerobic conditions for *P. freudenreichii* growing on lactate. Error bars represent standard error from biological replicates. Number of replicates per timepoint are displayed by circles (n = 2) and triangles (n = 3)

Biomass formation was found to increase 2.7-fold in microaerobic conditions in MM-lac compared to anaerobic conditions (Fig. 1A). In anaerobic conditions lactate was metabolised to propionate and acetate in a molar ratio of 1.98:1, close to the theoretical value of 2:1 (data not shown). In microaerobic conditions lactate was metabolised to propionate, acetate and pyruvate (Fig. 2). Contrary to the results of Dank et al. [9] obtained in rich medium, in our chemically defined medium the production of pyruvate was observed and propionate production declined. Biomass formation for anaerobic and microaerobic conditions did not differ significantly (0.38 vs 0.52 g CDW/L, independent student’s t-test (p = 0.38)) for cells growing on lactate at 48 h. When lactate was depleted no further biomass formation was observed in anaerobic conditions. In microaerobic conditions depletion of lactate was followed by oxidation of pyruvate and propionate to acetate and ${\mathrm{CO}}_{2}$ and a significant (independent student’s t-test (p < 0.01)) further increase in biomass (0.70 g/L anaerobic vs 1.87 g/L microaerobic). Total biomass formation after oxidation of propionate and pyruvate thus increased 2.7 times compared to anaerobic conditions, in line with result of Dank et al. [9], who observed an increase of 2.4.

Oxidation of propionate and pyruvate obviously resulted in an energetic benefit as shown by the increase in biomass formation. The increase in biomass formation was accompanied by a large increase of the B_12_ titer ($\mu g$/L), see Fig. 1B. The B_12_ titer during lactate metabolism in microaerobic conditions (t = 48 h) was found to be similar to that in anaerobic conditions (independent student’s t-test (p = 0.89)). However, a further increase of B_12_ was observed in microaerobic conditions, whilst in anaerobic conditions the B_12_ yield was minimally increased. The B_12_ titer increased sixfold in microaerobic conditions compared to anaerobic conditions (p = 0.088, independent student’s t-test), which means cells in microaerobic conditions produced on average two times more B_12_. As shown in Fig. 1B and Fig. 2 this may be attributed mainly to the oxidation of propionate and pyruvate. Pyruvate serves as major intermediate metabolite in carbon metabolism and thus is expected to contribute to biomass production and potentially production of B_12_. Propionate however is considered the metabolic end-product of anaerobic fermentation of lactate in propionic acid bacteria and is not linked directly as major carbon source for the production of biomass and B_12_. Our results thus raised the question whether propionate would serve as a suitable carbon source for *P. freudenreichii* for biomass formation and production of B_12_ in microaerobic conditions.

### Propionate oxidation supports biomass formation and ${\mathbf{B}}_{12}$ production

**Fig. 3:**
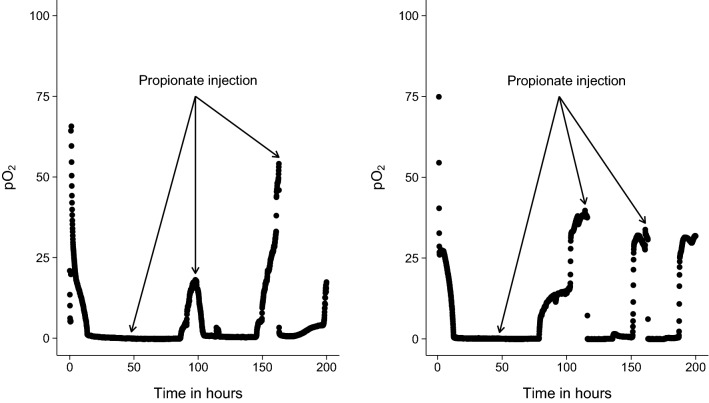
Dissolved oxygen measurement in bioreactors throughout cultivation on propionate. Dissolved oxygen as expressed as percentage of content measured at 100% air at 0.1 L/min at 30 ºC using 300 RPM and 0% air. Arrows indicate at which time new propionate was injected to an end concentration of 10 mM. Samples for biomass, HPLC and B_12_ quantification were taken directly prior to each new propionate injection

**Fig. 4:**
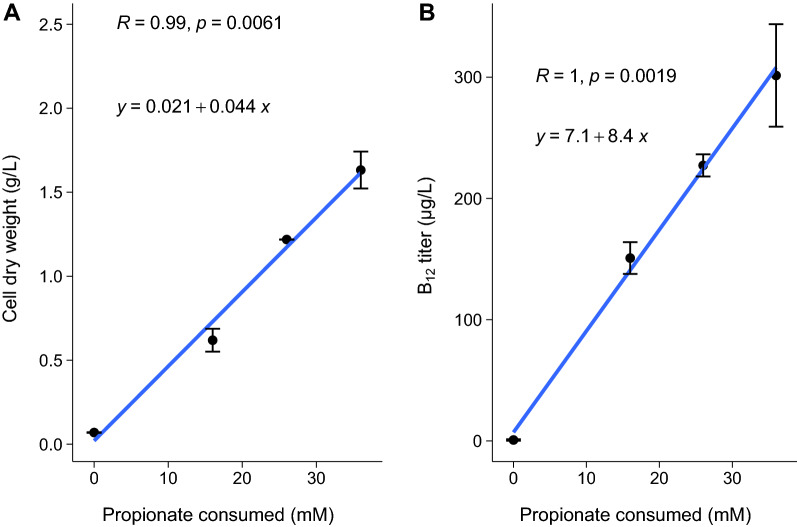
Biomass formation (**A**) and B_12_ titer (**B**) in microaerobic conditions for *P. freudenreichii* growing on propionate in a fed-batch reactor. Error bars represent standard error from biological duplicates

To study whether *P. freudenreichii* can grow on propionate as carbon source a bioreactor setup using minimal medium containing 100 mM propionate (MM-prop) was attempted. Surprisingly, no growth was observed under these conditions which may be attributed to a combination of inhibition by propionate [13], toxicity by oxygen [8] and low inoculum (see discussion). To minimise the product inhibition of propionate a fed-batch reactor was set up. Cells were pre-cultured in anaerobic conditions on lactate and transferred to a bioreactor with MM-propionate in microaerobic conditions. The inoculum size to the bioreactor was increased from 2% (v/v) to 10% (v/v). Propionate was fed to these cells to a final concentration of 10 mM at specific time points (t = 0 h, t = 48 h, t = 120 h and t = 168 h) whilst keeping the flux of oxygen constant. Injection of cells led to consumption of oxygen (Fig. 3) and complete consumption of propionate with a concomitant increase of biomass and B_12_ as shown in Fig. 4. The oxidation of propionate resulted first in the formation of acetate (data not shown). Oxygen was readily consumed after the primary injection and remained at the lower detection limit until depletion of both propionate and the formed acetate, after which oxygen levels steadily rose again. Injection of fresh propionate resulted in instantaneous consumption of oxygen, which confirmed respiratory pathways were used for metabolism of propionate and acetate with oxygen as terminal electron acceptor (Fig. 3). These results also indicate no loss of electron transport chain functionality at the oxygen fluxes used in our studies.

### Propionate is the substrate with the highest B_12_ yield per substrate and per biomass

**Fig. 5:**
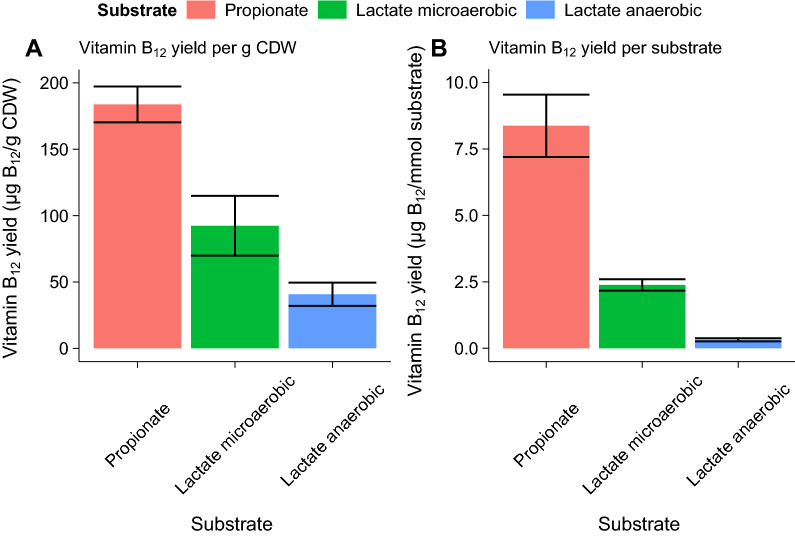
B_12_ yield per g cell dry weight (CDW) (**A**) and per mmol substrate (**B**) for *P. freudenreichii* in anaerobic and microaerobic conditions. Error bars represent standard error from biological replicates

The propionate fed-batch cultivation clearly shows the potential of propionate as a carbon source for B_12_ production. To compare the B_12_ yield on different substrates correctly the B_12_ yield per substrate was calculated at 168 h (Fig. 5). A 7.5-fold increased yield per substrate was found for microaerobic lactate-grown cells versus anaerobic grown cells. An increased yield of 26.3 times was found for microaerobic propionate-grown cells versus anaerobic lactate-grown cells. The yield per CDW increased two-fold for propionate-grown microaerobic cells versus lactate-grown microaerobic cells and 4.5 times for lactate-grown anaerobic cells. Both the productivity per substrate (p < 0.01 for propionate as substrate, multiple linear regression) and productivity per cell biomass (p < 0.05 for propionate as substrate, multiple linear regression) thus increases drastically when metabolising propionate in microaerobic conditions compared to lactate in anaerobic and microaerobic conditions.

## Discussion

*P. freudenreichii* has been extensively studied as a producer of B_12_ as it favours production of the human active form of B_12_ [14] and has the generally recognised as safe status [11].

Many different strategies for increasing B_12_ yield by *P. freudenreichii* have been attempted, such as genetic engineering [15], genome shuffling [16], media optimalisations [17] and changing environmental conditions such as presence or absence of oxygen [5] or activation of riboswitches using blue light [18]. In anaerobic processes the production of propionic acid (and conceivably acetic acid [19]) by *P. freudenreichii* causes product inhibition, resulting in decreased cell growth and reduced B_12_ synthesis [5, 20]. However, a decreased B_12_ yield per gram cells has also been reported for processes removing propionic acid efficiently [21]. Indeed, Wang et al. [20] have found maintaining propionic acid concentrations at specific levels can increase B_12_ production. The role and effect of propionic acid on final B_12_ yield thus remains complex, but points towards higher production of B_12_ with minimal presence of propionate in the environment. What most studies have in common is a goal to remove propionic acid (and acetic acid) or decrease its negative effect on cell growth in anaerobic conditions. Usually, this is done by mechanical means such as removing effluent whilst returning or immobilizing cells [22, 23]. Here we attempt to solve this problem in a bioenergetically favourable way; removal of propionic acid (and acetic acid) by oxidation, resulting in ATP generation and lower inhibition potential and possible activation of pathways requiring B_12_, such as the Wood-Werkman cycle.

Results obtained in the current study support the findings of Dank et al. [9] in chemically defined lactate medium in microaerobic conditions and show that during these conditions biomass production and B_12_ production drastically increases. However, contrary to Dank et al. [9] we also observed accumulation of pyruvate. Similar observations were found for *Acidipropionibacterium acidipropionici* by van Gent-Ruijters et al. [24], who attribute the accumulation of pyruvate to a lack of oxidative decarboxylation of pyruvate. Indeed, oxygen inhibits pyruvate-ferredoxin oxidoreductase (PFOR) [25, 26], which has been proposed to be a key enzyme during the utilisation of lactate by propionic acid bacteria [27]. Alternatively, in microaerobic conditions pyruvate may be directly oxidised using oxygen as acceptor by pyruvate oxidase (PO), producing CO_2_, H_2_O_2_ and acetyl-phosphate [27]. Consequently if the anaerobic route for pyruvate dissimilation is disabled due to inactive PFOR, accumulation of pyruvate can be expected when oxygen contents are limited and pyruvate dissimilatory pathways requiring oxygen directly (PO) or indirectly (through oxygen-dependent NADH:dehydrogenase activity (pyruvate dehydrogenase)) are limited in flux and compete with other processes requiring regeneration of NADH to NAD^+^. This hypothesis is supported by the observations of Ye et al. [28], who reported accumulation of pyruvate after injection of propionate in microaerobic conditions, implying the rate-limiting step during propionate oxidation to acetate occurs at the pyruvate node. Therefore, the most likely explanation is a stochiometric limitation of oxygen, limiting the amount of oxygen available for NADH dehydrogenase-coupled electron transport activity in combination with potential competition for oxygen by pyruvate oxidase and (partial) inactivation of other key metabolic enzymes such as PFOR, resulting in small NAD^+^ pools and pyruvate accumulation and production of propionate. The described stochiometric limitations in oxygen levels are in line with the reported sensitivity of *P. freudenreichii* to oxygen, while efficient substrate metabolism is supported in microaerobic conditions.

In our study propionate oxidation in microaerobic conditions resulted in a boost of B_12_ production, while in previous studies Ye et al. [5] observed ceased *B*_*12*_ production conceivably due to loss of $\delta$-aminolevulinic acid dehydratase activity [7] and consequently loss of cytochrome synthesis [8] in the high oxygenation conditions used in their experiments. Our results suggest it is key to keep oxygen fluxes low in order to maintain the ability to oxidise substrates using oxygen as a terminal electron acceptor. These results are supported by results of Tangyu et al. [29], who report highest B_12_ production at specific oxygen regimes in their food product. Since oxygen is required as terminal electron acceptor for oxidation of propionate, constraining oxygen to low levels results in oxygen being the growth rate determining factor. Since oxygen is supplied at a constant rate, the observed growth of *P. freudenreichii* on propionate in the microaerobic conditions used is linear [9]. Hence, to increase biomass formation and B_12_ production in time, higher oxygen fluxes should be applied, which requires increasing aerotolerance and/or respiration rates in *P. freudenreichii.* It is therefore interesting to attempt to obtain mutants with increased respiration rates by genetic engineering or adaptive evolution approaches. We hypothesised that the utilisation of propionate as sole carbon source for *P. freudenreichii* in microaerobic conditions will result in forcing flux through the reversed Wood-Werkman cycle. This in turn will result in a demand for B_12_ in growing cells as co-factor in the methylmalonyl-CoA transferase reaction and consequently activation of B_12_ production. However, in our first setup using 100 mM propionate in combination with the same microaerobic conditions applied on lactate no growth was observed. Both propionate [13] and oxygen [8] are known to be toxic for *P. freudenreichii*. Since the same microaerobic conditions were used as in the experiments on lactate as a carbon source, oxygen itself is not deleterious enough to inhibit growth at the used oxygen regime. Furthermore, Dank et al. [9] have shown that propionate is oxidised at higher concentrations (~ 70 mM) when larger amounts of biomass are present and pO_2_ inside the system is 0. Initial cell numbers are also reported to influence the potential of *P. freudenreichii* to either grow or not grow in milk [30]. Environmental stresses limit microbial growth in either synergistic or even multiplicative manner [31, 32]. Hence, it is conceivable the imposed stress of both propionate and oxygen in our initial setup was too big of a ‘hurdle’ for the low inoculum used in our study. It is therefore key to minimise the imposed stresses on *P. freudenreichii* by using a combination of low oxygen fluxes, low propionic acid concentrations and high initial biomass numbers.

Using a fed-batch system and thus low concentrations of propionate (10 mM), thereby preventing excessive product inhibition, we provide evidence that oxidation of propionate leads to production of acetate, which was further oxidised to CO_2_ during prolonged incubation. The oxidation of propionate to acetate and consequently to $C{O}_{2}$ leads to a significant increase of B_12_ production per cell biomass, i.e., an increased yield per g CDW of 184 $\mu g$/g CDW on propionate versus 92 $\mu g$/g CDW on lactate microaerobically and 41 $\mu g$/g CDW on lactate anaerobically. The presence of propionate as sole carbon source thus increased the yield considerably. To conclude, we have shown that propionate can serve as excellent carbon source for *P. freudenreichii* in microaerobic conditions. This opens up great potential for the application of microaerobic conditions in combination with controlled propionate feeding for efficient production of B_12_. Applications of (genetically) engineered B_12_-overproducing strains [15, 16] in combination with other optimisation strategies such as media optimisations [17] by addition of precursors or altering other environmental conditions such as light [18] as previously suggested are recommended to investigate further yield increments using propionate as substrate in microaerobic conditions. Further studies about the regulatory role of propionate in activation of B_12_ biosynthesis pathways in *P. freudenreichii* can provide clues for further optimisation.

## Conclusions

Here we show minimal fluxes of oxygen can greatly enhance biomass and B_12_ production in *P. freudenreichii* with lactate or propionate as a substrate. Stochiometric constraints of oxygen cause triauxic growth on lactate as observed earlier by Dank et al. [9]. The formation and subsequent oxidation of propionate appeared to be linked to increased B_12_ titer and yield. Fed-batch experiments showed that propionate can serve as excellent carbon source for biomass production and B_12_ production. Further studies on the potential regulatory role of propionate in activation of B_12_ synthesis in *P. freudenreichii* need to be performed. Since the oxidation of propionate is limited by the stochiometric constraint of oxygen, optimizing oxygen fluxes, increasing aerotolerance and/or respiration rates in *P. freudenreichii* may aid in improving oxidation rates and thus biomass and B_12_ production.

## Methods

### Strain and preculture conditions

*P. freudenreichii* DSM 20271 was obtained from Deutsche Sammlung von Mikroorganismen und Zellkulturen (DSMZ) and routinely grown on yeast extract lactate (YEL) consisting per liter of: 10 g tryptone, 5 g yeast extract, 5 g $K{H}_{2}P{O}_{4}$ and 16 g 80% l-Lactate syrup (Sigma Aldrich) and 15 g bacteriological agar for plates. Cell cultures were grown for 3 days in liquid media and maintained in 30% (v/v) glycerol stocks at −80 °C. Cells were precultured for each experiment by streaking *P. freudenreichii* on YEL agar and incubating at 30 °C in anaerobic conditions for 7 days. Single colonies were inoculated in minimal medium with composition described below.

### Minimal media

Minimal media (MM) used in this study consisted per liter of: 100 mM carbon l-lactate (MM-lac), 10 mL metal stock(100x), 10 mL nucleic acid stock(100x), 10 mL vitamin stock(100x) and 400 mL amino acids stock(2.5x) with the following compositions for each stock described below. Metal stock per kg: $MgC{l}_{2}.6{H}_{2}O$ 20 g, $CaC{l}_{2}.2{H}_{2}O$ 5 g, $ZnS{O}_{4}.7{H}_{2}O$ 0.5 g, $CoC{l}_{2}.6{h}_{2}O$ 0.25 g, $MnC{l}_{2}.4{H}_{2}O$ 1.6 g, $CuS{O}_{4}.5{H}_{2}O$ 0.25 g, $\left(N{H}_{4}\right)6M{o}_{7}{O}_{24}.4{H}_{2}O$ 0.25 g, $FeC{l}_{3}.6{H}_{2}O$ 0.3 g, $FeS{O}_{4}.7{H}_{2}O$ 0.3 g ($FeS{O}_{4}.7{H}_{2}O$ was first dissolved in 10 ml 17% HCl, before it was mixed with the other compounds). Nucleic acid stock per kg: 1 g of each dissolved in 0.1 M NaOH; adenanine, uracil, xanthine, guanine. Vitamin stock per kg: Ca-d-pantothenate 0.1 g, d-biotin 0.25 g, thiamin-HCl 0.1 g, na-p-aminobenzoate 1 g. Amino acids stock: 1 mM of l-Alanine, l-Arginine Hydrochloride, l-Asparagine monohydrate, l-Aspartic Acid, l-Cysteine hydrochloride, l-Cystine, l-Glutamic Acid, l-Glutamine, Glycine, l-Histidine hydrochloride, l-Isoleucine, l-Leucine, l-Lysine hydrochloride, l-Methionine, l-Proline, l-Serine, l-Threonine, l-Tryptophan, l-Tyrosine, l-Valine. For fed-batch experiments l-lactate was replaced by 10 mM propionate (MM-prop).

### Bioreactor cultivations on lactate

Bioreactor cultivations were performed according to the methods described by [9]. A single colony of *P. freudenreichii* was inoculated from YEL agar plates in 10 mL MM-lac and incubated at 30 °C anaerobically for 5 days, after which 2% (v/v) was inoculated into bioreactors with a working volume of 500 mL (Multifors, Infors HT, Switzerland). The stirring speed was set at 300 RPM, the temperature was kept constant at 30 °C and the pH was controlled at 7.0 by automatic addition of 5 M NaOH and 1 M HCl. A gas mix containing 85% ${N}_{2}$ gas and 15% air was used for microaerobic conditions. Gas was supplied through a sparger at the bottom of the fermenter using a mass flow controller premixing gas at set values at a rate of 0.1 L/min. Dissolved oxygen was measured using a probe which was calibrated at 100% by flushing the system with pure air at 0.1 L/min for 2 h and at 0% by flushing the system with ${N}_{2}$ for 2 h. Samples were taken at various time points aseptically through a sampling port. *P. freudenreichii* was grown in anaerobic conditions in 50 mL greiner tubes in MM-lac as described above and sampled at the several timepoints as reference condition. All samples were stored at −20 °C.

### Fed-batch cultivations on propionate

*P. freudenreichii* was precultured on MM-lac in anaerobic conditions as described before. *P. freudenreichii* was inoculated into bioreactors containing 10 mM propionate minimal medium (MM-prop). Bioreactor settings were equal to settings used for cultivation on lactate described above. 10 mL samples were taken at 0 h, 48 h, 120 h and 168 h. After each sample point a new injection with 10 mL of 500 mM propionate stock was made to establish an end concentration of 10 mM propionate in the reactor after the injection. All samples were stored at −20 °C.

### Biomass quantification

Biomass was quantified by measuring the cell dry weight (CDW) concentration as described by van Mastrigt et al. [33]. Membrane filters with a pore size of 0.2 µm (Pall Corporation, Ann Arbor, MI, USA) were pre-dried in an oven at 80 °C and then weighed. Samples were passed through the pre-weighted membrane filters using a vacuum filtration unit. Residual cell material in the funnel was washed to the filter using approximately 30 mL of demi water. The filters containing the biomass were dried at 80 °C again, after which filters were weighed again. CDW was calculated using the following formula:

$$CDW (\frac{g}{kg})= \frac{Weight\,filter+biomass \left(g\right)-weight\,filter(g)}{Amount\,of\,culture (g)}*1000$$

### Analysis of organic acids

Lactate, acetate, propionate and pyruvate were quantified by High Performance Liquid Chromatography (HPLC) as described by van Mastrigt et al. [34]. Briefly, 500 µL of sample was deproteinized by addition of 250 µL Carrez A (0.1 M potassium ferrocyanide trihydrate), mixing, addition of 250 µL Carrez B (0.2 M zinc sulfate heptahydrate), mixing and centrifugation for 2 min at 17,000 × g. 200 µL supernatant was injected on a UltiMate 3000 HPLC (Dionex Germany) equipped with an Aminex HPX-87H column (300 × 7.8 mm) with guard column (Biorad). 5 mM H_2_SO_4_ was used as mobile phase with a flow rate of 0.6 mL/min at a column temperature of 40 °C. Compounds were detected using a refractive index detector (RefractoMax 520).

### B_12_ quantification

B_12_ was detected using a Vitafist B_12_ biological assays (R-Biopharm). Samples were prepared for analysis by diluting them 10 × with demi water. Samples were beat-beaded (lysing matrix B, mp-bio) in FastPrep-24 instrument (MP Biomedicals) 3 times using 1-min intervals followed by centrifugation at 17,000 × g for 2 min. Supernatants were collected and diluted 4 × with demi water, after which they were heated for 30 min at 95 °C in a water bath. Samples were chilled on ice and diluted further to fall within the microbiological assay detection range. B_12_ assays were performed as described by the manufacturers protocol in technical replicates. Absorbance was measured in microtiter plates using Microwell Plate Reader SpectraMax M2 at 610 nm with SoftMax Pro software.

### Statistical analysis

Statistical analysis was performed using R in combination with Rstudio. Data normality was tested using Shapiro-Wilk test. Equal or unequal variance was tested using F-tests. Both normality and equal variances were assumed when p > 0.05. Independent-students t-tests were used based on equal variances using R t.test function. The effects of microaerobic conditions and propionate as substrate were estimated using multiple linear regression in R using the lm function. Both yield per substrate and per cell were fitted using substrate and condition as dependent variables.

## Supplementary Information

**Additional file 1.** Datasheet containing all data gathered and used for this study.

## Data Availability

The dataset supporting the conclusions of this article is included within Additional file 1.

## Abbreviations

PFOR: Pyruvate: ferredoxin oxidoreductase
PO: Pyruvate oxidase
*P*: *Propionibacterium*
CDW: Cell dry weight
Vitamin B_12_: B_12_
MM: Minimal medium
DSMZ: Deutsche Sammlung von Mikroorganismen und Zellkulturen
YEL: Yeast extract lactate
